# Nitidine chloride suppresses epithelial‐mesenchymal transition and stem cell‐like properties in glioblastoma by regulating JAK2/STAT3 signaling

**DOI:** 10.1002/cam4.3869

**Published:** 2021-03-31

**Authors:** Mingbo Jia, Ying Wang, Yingxue Guo, Pengyue Yu, Ying Sun, Yanke Song, Liyan Zhao

**Affiliations:** ^1^ Department of Clinical Laboratory The Second Hospital of Jilin University Changchun China

**Keywords:** apoptosis, epithelial‐mesenchymal transition, glioblastoma, gliosphere, JAK2/STAT3, nitidine chloride

## Abstract

Glioblastoma is the most aggressive and common intracranial malignant tumor, and the prognosis is still poor after various treatments. Based on the poor prognosis of glioma, new drugs that suppress the rapid progression and aggressive growth of glioma are urgently needed. It has been reported that nitidine chloride (NC) can inhibit tumor growth and epithelial‐mesenchymal transition (EMT), and EMT is associated with cancer stem cell properties. The present study aimed to investigate the inhibitory effect of NC on the EMT process and stem cell‐like properties in glioma cells. The results showed that the migration and invasion abilities in U87 and LN18 glioma cells were significantly increased after the induction of EMT and these effects were inhibited by NC in a concentration‐dependent manner. NC treatment decreased the expression of EMT markers in glioma cells and self‐renewal capacity of glioma stem‐like cells. We demonstrated that these effects of NC were achieved via JAK2/STAT3 signaling. Taken together, these results indicate that NC inhibits the EMT process and glioma stem‐like properties via JAK2/STAT3 signaling pathway, suggesting that NC may be a potential anti‐glioma drug.

## Graphical abstract

NC can inhibit the EMT process and glioma stem cell characteristics of glioma cells via modulation of JAK2/STAT3 signaling, suggesting that NC may be a potential anti‐glioma drug.

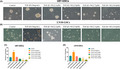

## 1 INTRODUCTION

Glioblastoma is the most common and malignant intracranial tumor in adults. Glioblastoma is associated with a rapid invasive rate, a high recurrence rate after surgery, a poor prognosis, and a low survival rate. These characteristics may be related to the epithelial‐mesenchymal transition (EMT) process and stem cell properties of glioma cells. The previous studies have shown that through EMT, cells achieve an altered phenotype with high migration and invasion abilities, and thus, EMT is the critical step in the malignant progression of tumors. These cells serve to enrich the glioma stem cells, which may promote the invasion and recurrence of gliomas.^1^, ^2^, ^3^, ^4^ Glioblastomas contain a small subset of cells with stem cell characteristics that have self‐renewal ability. Glioma stem cells have been linked to radiotherapy and chemotherapy resistance of glioblastoma. In the process of tumor metastasis activated by EMT, diffuse tumor cells exhibit self‐renewal capacities similar to stem cells. This increases the EMT process and the self‐renewal ability of cancer cells, making the cancer cells more aggressive.^5^, ^6^ Cancer stem cells can enter the bone marrow and circulation and migrate to secondary tissue sites, similar to mesenchymal cells, while proliferating, differentiating, and participating in tumor reconstruction.^7^ Therefore, inhibition of the EMT process of glioma cells in combination with therapy targeting glioma stem cells may be the key to successful treatment of glioblastoma. However, there is no drug that not only suppresses EMT of glioma cells, but also eliminates glioma stem cells.

Nitidine chloride (NC) is extracted from the roots of Rutaceae plants and the stems of *Zanthoxylum bungeanum Maxim*. NC was first found to have an obvious inhibitory effect on reverse transcriptase and DNA polymerase activities in mice, and later a prominent anti‐tumor role for NC was discovered.^8^ Studies have shown that NC can inhibit the growth of tumor cells in many kinds of tumors.^9^, ^10^ Not only that, but also alleviate the resistance of chemoradiotherapy. Although NC can affect the activity of multiple signaling pathways, the inhibitory effect of NC on the JAK2/STAT3 signaling pathway was found to be prominent.^11^, ^12^, ^13^, ^14^, ^15^ However, the action of the JAK2/STAT3 signaling pathway in glioma cell EMT and glioma stem cells has not been fully elucidated.

The JAK2/STAT3 signaling pathway is associated with a malignant progression of tumor cells, such as cell proliferation, invasion, and immunoregulation. JAK2/STAT3 signaling plays an important role in the malignant progression of tumors.^16^ In cervical cancer, the EMT process of cancer cells was regulated through the JAK2/STAT3 signaling pathway.^17^ In non‐small cell lung cancer, mesenchymal stem cells were shown to promote the formation of cancer stem cells through the IL6/JAK2/STAT3 pathway and to promote tumor invasion and migration.^18^ In radiotherapy‐resistant advanced colorectal cancer tissues, the JAK2/STAT3 signaling pathway plays a role in promoting tumorigenesis and drug resistance by inhibiting cell apoptosis and enhancing cell cloning potential. Moreover, the combination of STAT3 and cyclin D2 promoter promotes the continuous growth of cancer stem cells.^19^ Therefore, a comprehensive understanding of the abnormal expression of members of the JAK2/STAT3 signaling pathway and the effects on the classic EMT process and cancer stem cells provide new ideas for the development of methods to effectively inhibit the malignant progression of glioma.

In the present study, we demonstrated the effects of NC on the EMT process in glioma cells and the activities of glioma stem‐like cells. The results showed that NC inhibited the glioma EMT process and the properties of glioma stem‐like cells by modulating the JAK2/STAT3 signaling pathway, suggesting the potential of NC to serve as an effective anti‐glioma drug.

## 2 MATERIALS AND METHODS

### 2.1 Reagents and antibodies

NC was purchased from APExBIO. Anti‐cleaved caspase‐3, anti‐ Bax, anti‐B‐cell lymphoma (Bcl)‐2, anti‐Anti‐poly(ADP‐ribose) polymerase (PARP), anti‐cleaved PARP, anti‐E‐cadherin, anti‐Snail, anti‐Twist1, anti‐Bmi1, anti‐Slug, anti‐Sox2, anti‐Oct4, anti‐vimentin, anti‐β‐catenin, anti‐N‐cadherin, anti‐JAK2,anti‐STAT3, anti‐phosphorylated (p)‐STAT3, anti‐p‐JAK2 antibodies, and anti‐β‐actin were purchased from Cell Signaling Technology. Epidermal growth factor (EGF), basic fibroblast growth factor (bFGF), and transforming growth factor (TGF)‐β1 were purchased from PeproTech. N2 (100×), B27 (50×), and GlutaMAX was purchased from Gibco.

### 2.2 Cell culture

Human glioblastoma cell lines U87 and LN18 (U87 and LN18 glioma cells), were obtained from American Type Culture Collection (ATCC). The cells were cultured in a 37℃, 5% CO_2_ incubator.

### 2.3 Induction of EMT by TGF‐β1

EMT was induced by TGF‐β1. TGF‐β1 was added to U87 and LN18 glioma cells, the cells were cultured in a 5% CO_2_ incubator at 37℃ for 48 h.

### 2.4 Glioma U87 and LN18 cell culture

U87 and LN18 cell density was adjusted to (3×10^5^ cells/ml), and 2% B27, 20 ng/ml of bFGF, and 1% N2was added to the neurobasal medium. Gliosphere culture medium was prepared by the addition of 20 ng/ml of EGF, 1% GlutaMAX, and 100 U/ml of penicillin‐streptomycin, and 4 ml of culture medium was added to culture the spheroids in a 37°C, 5% CO_2_ incubator.

### 2.5 Gliosphere formation assay

The density of the first‐generation gliospheres was adjusted to 5×10^3^ cells/ml for plating of cells into 96‐well plates. Neurobasal medium containing 1% N2, 2% B27, 20 ng/ml of bFGF, 20 ng/ml of EGF, 1% GlutaMAX, and 100 U/ml of penicillin‐streptomycin was used for the gliosphere culture medium. Cells were treated with TGF‐β1 and NC at the indicated concentrations in 0.2 ml of medium per well and cultured for 7 days, with the addition of 0.05 ml of medium every 2 days. The numbers of gliospheres (>50 cells) in each well were then counted under a microscope.

### 2.6 Limiting dilution assay

The gliospheres were digested, and the cells were seeded in 96‐well plates. The cell dilutions were 20, 40, 60, 80, 100, 120, 140, 160, 180, and 200 cells in 0.2 ml of medium per well. The percentage of cells in each well that did not a form gliosphere was calculated for each inoculation density. A graph was constructed with the number of cells as the abscissa and the percentage of cells that did not form a gliosphere as the ordinate.

### 2.7 Cell proliferation assay

U87 and LN18 glioma cells (8×10^3^ cells/well) were seeded in 96‐well plates in a 0.2 ml of medium for gliosphere formation. After 24 h of culture, cells were treated with the indicated concentrations of NC dissolved in dimethyl sulfoxide (DMSO). An equal amount of DMSO was added to the control as the control group. After 24 and 48 h, 20 μl of CCK‐8 (Beyotime) reagent was added to each well, and the cells were incubated at 37℃ in 5% CO_2_ for 30 min before the measurement of the absorbance in each well at 450 nm for the calculation of the percentage of viable cells via the CCK‐8 assay.

### 2.8 Western blot analysis

After the treatment of U87 and LN18 glioma cells with different concentrations of NC for specific time periods, the cells were collected for western blot analysis. The antibodies for proteins used in this analysis were as described above. The protein samples were transferred to polyvinylidene difluoride (PVDF) membranes. The PVDF membranes were rinsed again and exposed to the enhanced chemiluminescence (ECL) color developing solution in darkness. Finally, the PVDF membranes were placed in a gel imaging analyzer (Sage Creation) for the collection of images for analysis.

### 2.9 In vitro scratch assay

U87 and LN18 glioma cells (1.5×10^5^ cells/well) were seeded in 6‐well plates for culture at 37℃ in a 5% CO_2_ incubator for 24 h. Three fields of vision were randomly selected in each well for the collection of 0 h scratch photographs. Images were taken again after 24 h and 48 h to measure the change in the scratch area and calculate the cell migration distance. The experiment was repeated three times, and the results were averaged.

### 2.10 In vitro invasion assay

Transwell plates were prepared by adding 60 μl of cooled Matrigel solution to each well and allowing it to solidify at room temperature for 8 h. The Matrigel‐coated membrane in each well was wetted prior to seeding the U87 and LN18 glioma cells (5×10^4^ cells/well) with a 200 μl of serum‐free medium. The number of invading cells was counted in each image, and the cell invasion rate was calculated as follows to express the invasive ability of the cells: Cell invasion rate (%) = number of invading cells in the NC treatment group/number of invading cells in the control group ×100%.

### 2.11 Flow cytometric analysis

U87 and LN18 glioma cells (2×10^5^ cells/well) were treated with NC at different concentrations, and then 5 μl of Annexin‐V fluorescein isothiocyanate (FITC) reagent and 10 μl of propidium iodide (PI) reagent were added to each sample for staining for 30 min in darkness. The ratio of apoptotic cells among the total number of cells was then detected by flow cytometry (Beckman Coulter).

### 2.12 Statistical analysis

GraphPad Prism 7.0 software was used for data analyses. Comparisons among multiple groups were performed by one‐way analysis of variance. Each experiment was repeated three times, and the results are expressed as means ±standard error. Differences for which *p*<0.05 were considered statistically significant.

## 3 RESULTS

### 3.1 NC inhibits the proliferation of glioma cells

**FIGURE 1:**
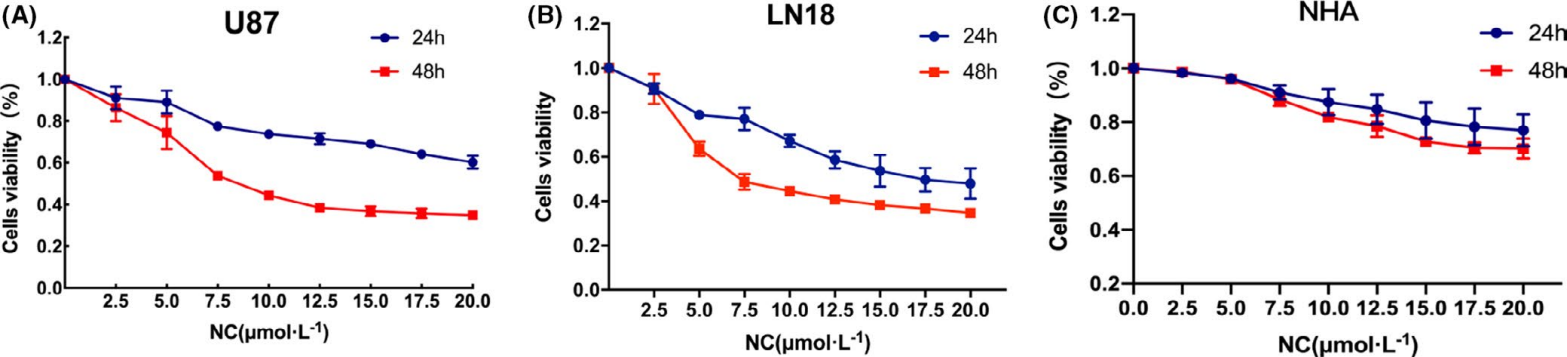
(A, B) NC inhibited the proliferation of glioma cells treated with NC for 24 h and 48 h. The viability of glioma U87 and LN18 cells was detected by the CCK‐8 method. C NC inhibited the proliferation of NHA cells treated with NC for 24 h and 48 h. The viability of NHA cells was detected by the CCK‐8 method. Independent experiments were repeated three times, and the results were compared with those for the control group

To determine the appropriate concentration of NC for inhibiting EMT of glioma cells, first, a CCK‐8 assay was used to detect the inhibitory effect of NC on U87 and LN18 glioma cells activity. After the treatment of U87 and LN18 glioma cells with NC (0, 2.5, 5.0, 7.5, 10.0, 12.5, 15.0, 17.5, and 20.0 μM) for 24 or 48 h, the survival rate of the cells was detected. With an NC concentration of 0 (control) or 2.5 μM, little effect on the cell survival rate was observed, with survival rates of approximately 80% at both 24 and 48 h. The IC_50_ concentration for NC was between 5.0 and 7.5 μM with a treatment time of 48 h. Therefore, the four NC concentrations of 2.5, 5.0, 7.5, and 10.0 μM were selected for subsequent experiments. These NC concentrations had a significant inhibitory effect on the proliferation of glioma cells, and this inhibitory effect became more obvious with increasing NC concentration (Figure 1A and B).

In order to observe the toxic effect of NC on non‐tumorigenic brain cell lines, experiments were carried out with NHA cells. The results showed that the survival rate of NHA cells was not significantly reduced when the cells were treated with different concentrations of NC for 24 and 48 h, and the IC_50_ values in NHA cells were much smaller than those in U87 and LN18 cells. In contrast, the toxicity of NC to NHA cells was limited (Figure 1C).

### 3.2 TGF‐β1 induces EMT in glioma cells

**FIGURE 2:**
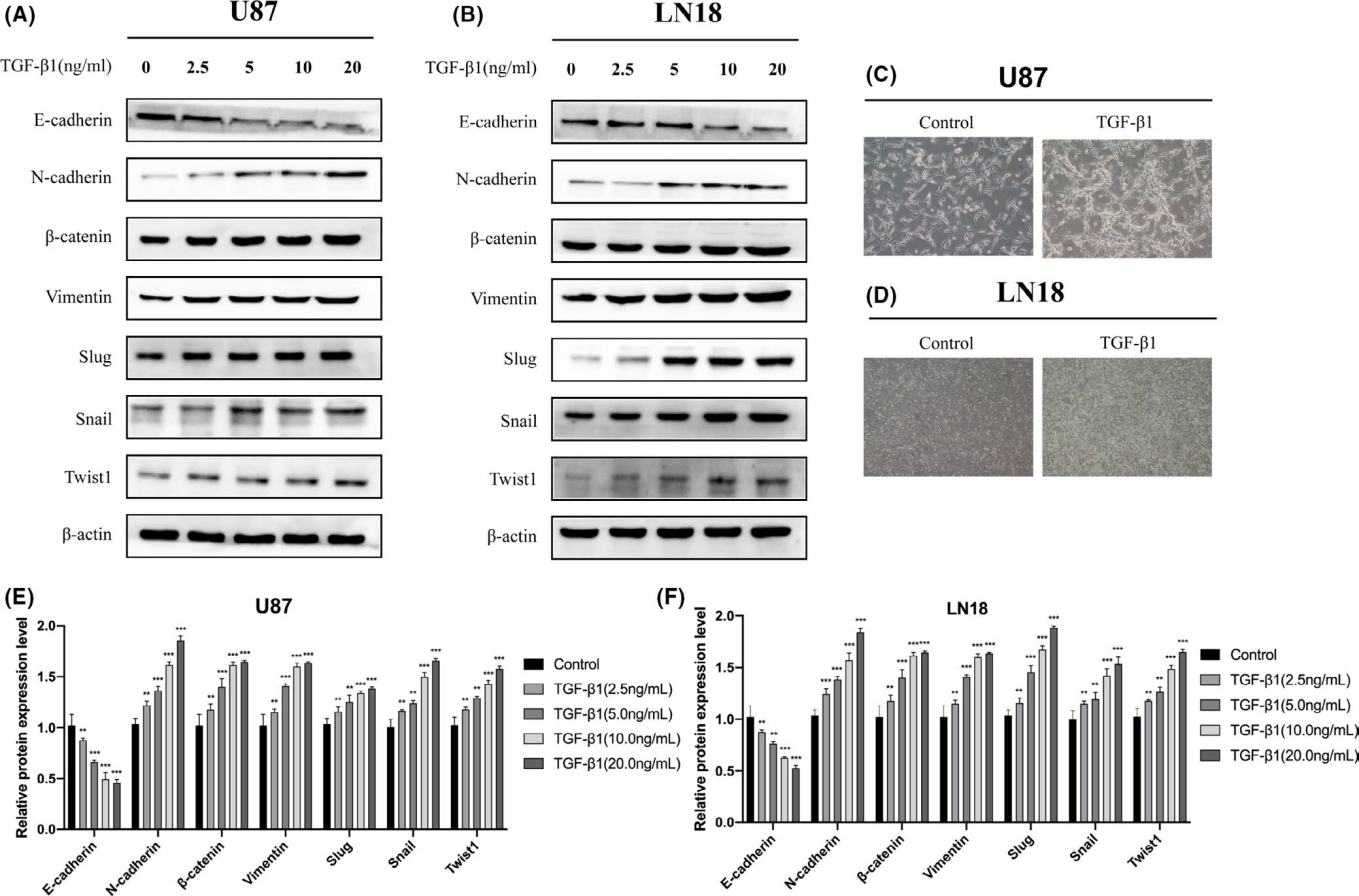
(A, B) Glioma U87 and LN18 cells were treated with TGF‐β1 for 48 h to induce EMT. Western blotting analysis was performed to detect the expression of EMT‐related protein markers as well as β‐actin as an internal reference. (C, D) Microscopic images showing the morphology of U87 (100x) and LN18 (100x) cells after treatment with TGF‐β1 (10 ng/ml) for 48 h. (E, F) Quantitative analysis of the protein levels, Data were obtained from three independent experiments. *p<0.05, **p<0.001, ***p<0.0001

TGF‐β1 was found to induce EMT in U87 and LN18 glioma cells based on the expression of different protein markers. As expected during EMT, the expression level of epithelial marker E‐cadherin was reduced, while those of the mesenchymal markers N‐cadherin, vimentin, and β‐catenin were increased. Moreover, the increased expression levels of Snail, Slug, and Twist1 also indicated an increase in EMT among the treated cells (Figure 2).

### 3.3 NC suppresses EMT of glioma cells

**FIGURE 3:**
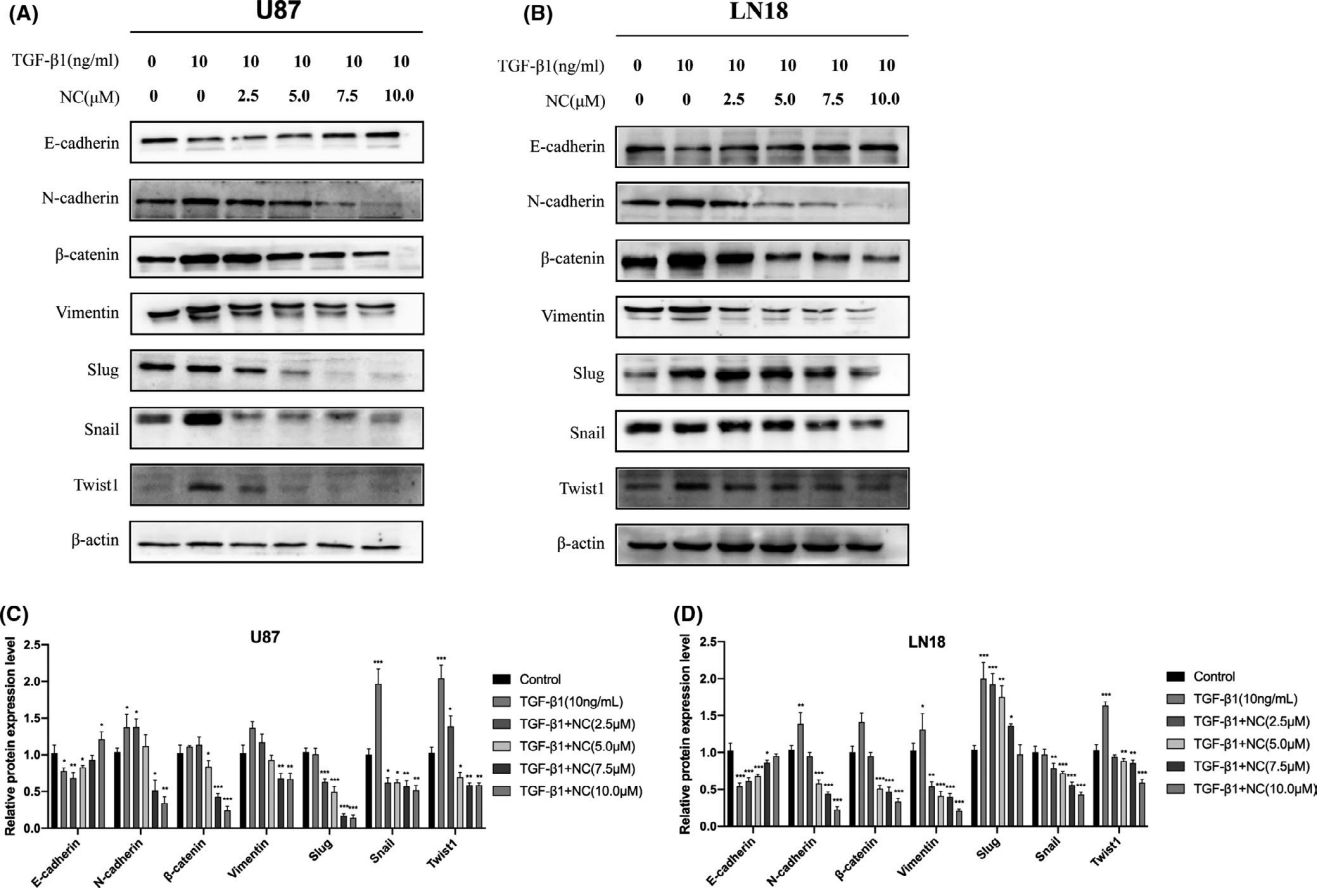
(A, B) EMT was induced in glioma cells by treatment with TGF‐β1 (control cells were not treated with TGF‐β1). Then the cells were treated with different concentrations of NC (2.5, 5.0, 7.5 and 10.0 μM) for 48 h. Western blotting analysis was performed to detect the expression of EMT‐related protein markers as well as β‐actin as an internal reference. (C, D) Quantitative analysis of the protein levels, Data were obtained from three independent experiments. *p<0.05, **p<0.001, ***p<0.0001

To explore the inhibitory effect of NC on EMT in glioma cells, we tested the influence of NC on the expression of important epithelial and mesenchymal markers as well as transcription factors during EMT of glioma cells. The experimental results showed that after the induction of EMT in U87 and LN18 glioma cells, treatment with different concentrations of NC inhibited the expression of relevant marker proteins to different degrees. Treatment with NC at concentrations greater than 7.5 μM significantly upregulated the expression of epithelial marker E‐cadherin. The expression levels of vimentin, Snail, N‐cadherin, β‐catenin, Slug, and Twist1 were downregulated. These results indicated that NC had a significant inhibitory effect on the EMT of U87 and LN18 glioma cells, and this inhibitory effect was concentration‐dependent (Figure 3A and B).

### 3.4 NC inhibits the migration and invasion of glioma cells

**FIGURE 4:**
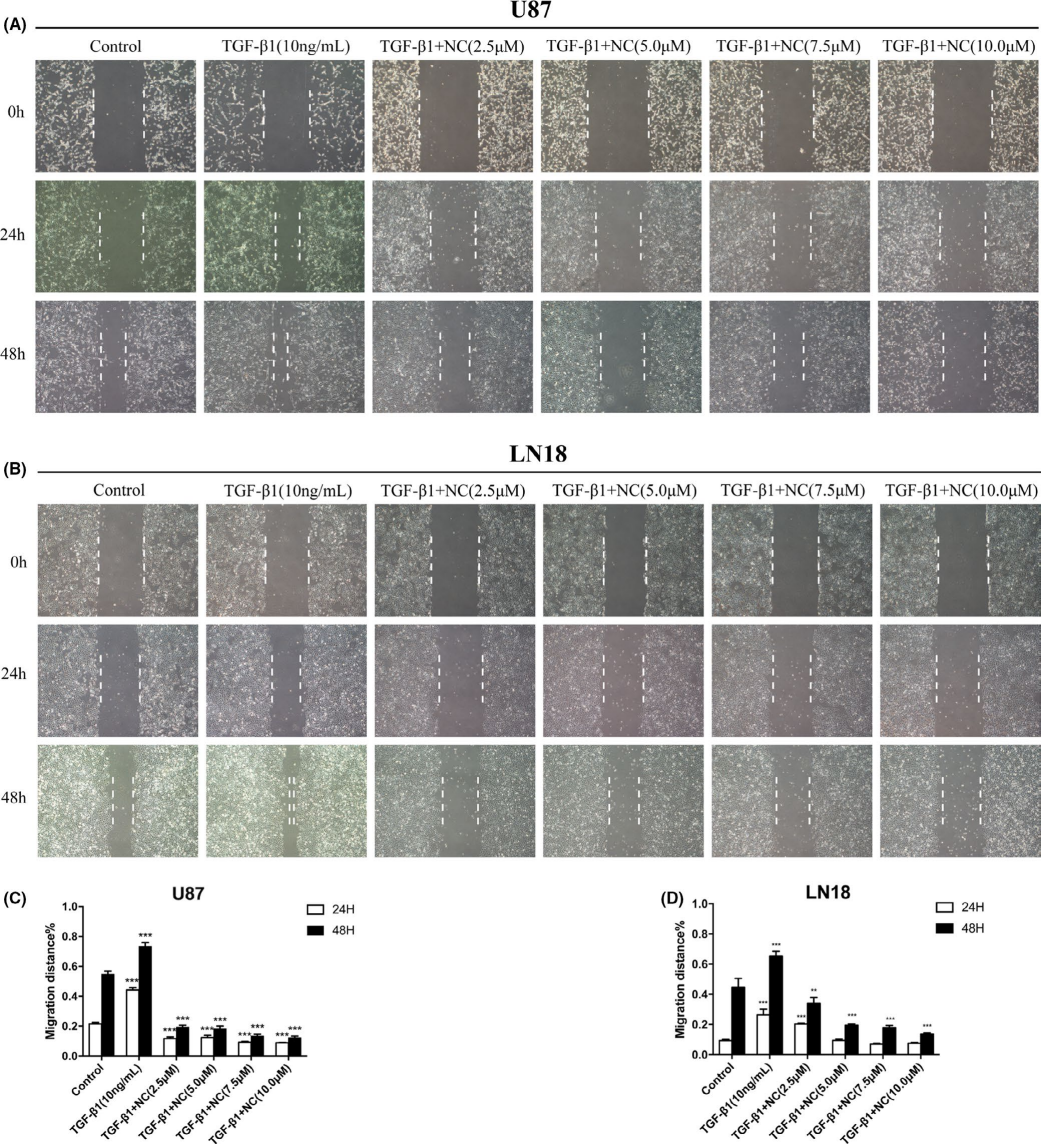
(A, B) U87 and LN18 glioma cells were treated with TGF‐β1 to induce EMT, and then the migration the cells was analyzed by a scratch assay after treatment with NC (2.5, 5.0, 7.5 and 10.0 μM). Microscopic images (200x) were taken after 24 and 48 h of NC treatment. (C, D) Quantitative results for glioma cell migration after induction of EMT and treatment with NC. Each independent experiment was repeated three times, and the results were compared with the those for the control group. *p<0.05, **p<0.001, ***p<0.0001

After finding that NC inhibits EMT in glioma cells, we further investigated whether NC has an effect on glioma cell migration and invasion induced by EMT. The results revealed that the scratch width in the EMT group was significantly shorter than that in the control group, and the cell migration rate was also obviously increased, indicating that the induction of EMT enhanced the migration of U87 and LN18 glioma cells. However, treatment of the cells with NC increased the scratch width and reduced the cell migration rate in a concentration‐dependent manner, indicating that NC can reduce the migratory of glioma cells. Overall, these results indicated that NC reduced the enhanced migration of glioma cells that occurs following EMT (Figure 4A–D).

**FIGURE 5:**
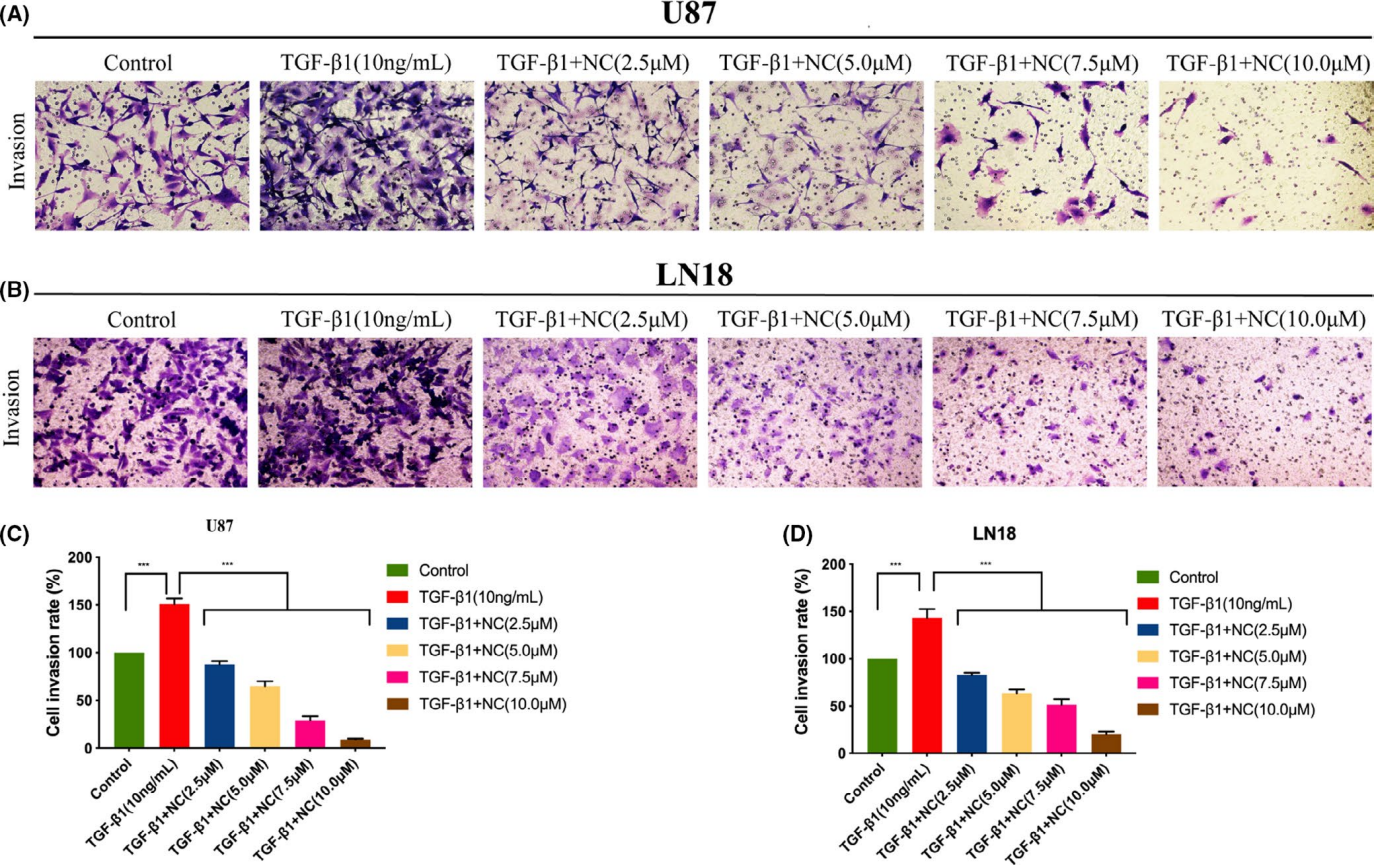
(A, B) U87 and LN18 glioma cells were treated with TGF‐β1 to induce EMT, and then the invasive ability of the cells was analyzed by Transwell assay after treatment with NC (2.5, 5.0, 7.5 and 10.0 μM). Microscopic images (200x) were taken after 24 and 48 h of NC treatment. (C, D) Quantitative results for glioma cell invasion after induction of EMT and treatment with NC. Each independent experiment was repeated three times, and the results were compared with the those for the control group. *p<0.05, **p<0.001, ***p<0.0001

To investigate the influence of NC on the invasive ability of glioma cells after EMT, we used a Transwell invasion assay. The experimental results showed that compared with the blank control group, the number of cells penetrating the membrane in the EMT group was significantly increased along with the cell invasion rate, whereas in the groups treated with NC, fewer cells penetrated the membrane, and the cell invasion rate was also markedly reduced in a concentration‐dependent manner. These results indicated that NC significantly decreased the invasive ability of glioma cells after EMT (Figure 5A–D).

### 3.5 NC promotes glioma cell apoptosis

**FIGURE 6:**
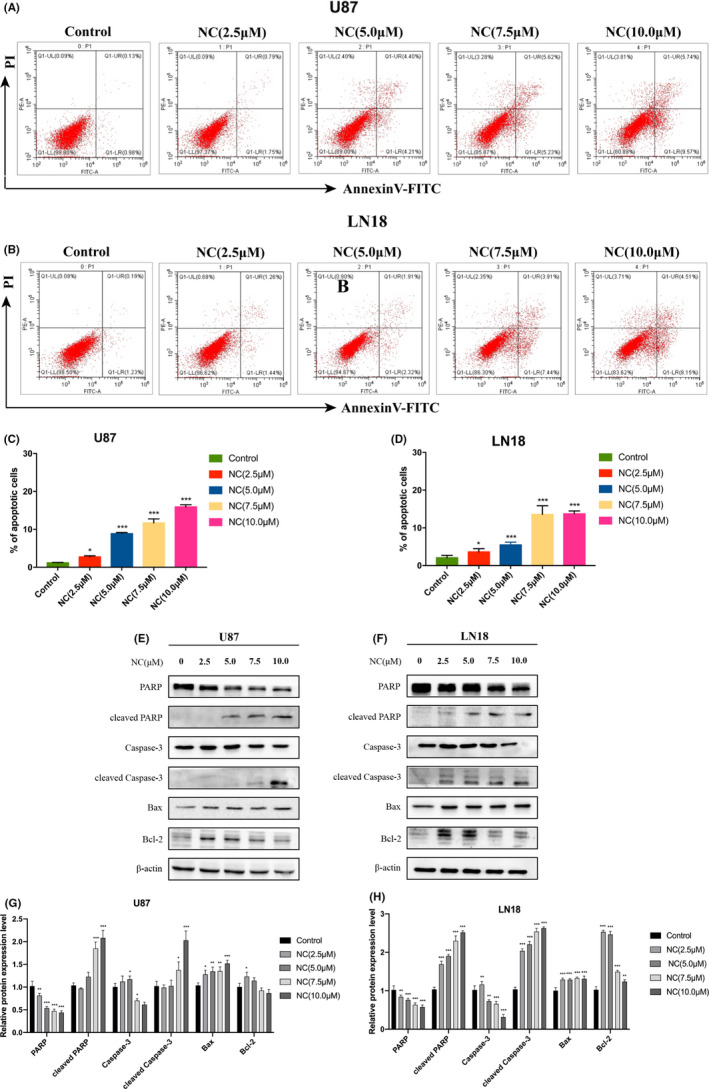
(A, B) U87 and LN18 glioma cells were treated with NC (2.5, 5.0, 7.5 and 10.0 μM) for 48 h, and then apoptotic cells were detected by flow cytometry. (C, D) Quantitative data results from flow cytometry. Each independent experiment was repeated three times, and the results were compared with those for the control group. *p<0.05, **p<0.001, ***p<0.0001. (E, F) U87 and LN18 glioma cells were treated with NC (2.5, 5.0, 7.5 and 10.0 μM) for 48 h, and then the expression levels of apoptosis‐related proteins were detected by western blotting. (G, H) Quantitative analysis of the protein levels, Data were obtained from three independent experiments. *p<0.05, **p<0.001, ***p<0.0001

To determine the mechanism by which NC inhibits the proliferation of glioma cells, the apoptotic rate among glioma cells was detected by the Annexin‐V FITC and PI double staining method and flow cytometry. The results revealed that NC promoted apoptosis among glioma cells in a concentration‐dependent manner (Figure 6A–D). In addition, we also performed western blotting to detect the expression of apoptosis‐related proteins. NC treatment reduced the expression of caspase‐3, PARP, and Bcl‐2, while increasing the expression of the apoptotic proteins cleaved caspase‐3, cleaved PARP, and Bax. These results indicated that NC promoted the apoptosis of glioma cells based on the increased expression of apoptosis‐related proteins (Figure 6E and F).

### 3.6 NC inhibits JAK2/STAT3 signaling in glioma cells

**FIGURE 7:**
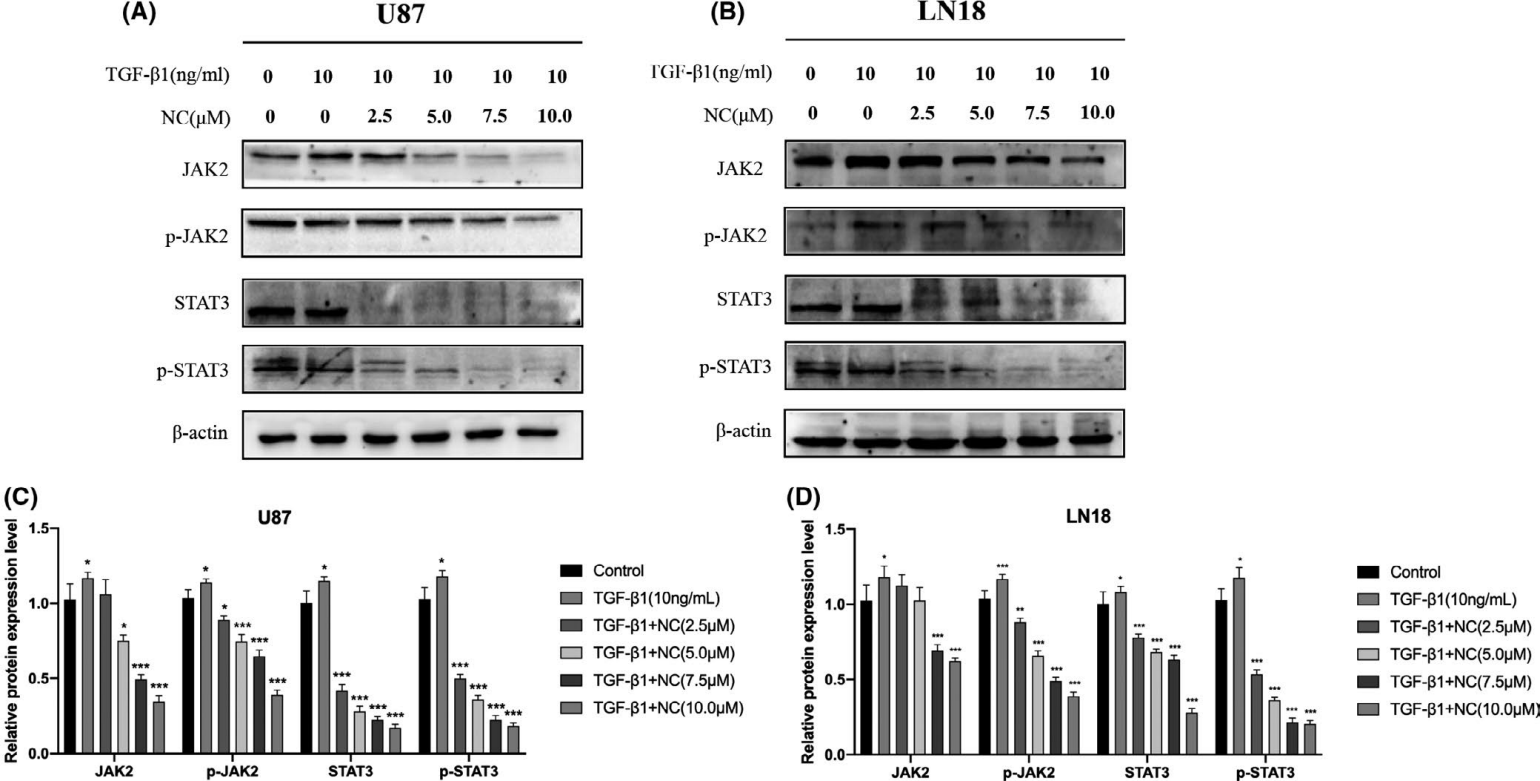
(A, B) U87 and LN18 glioma cells were treated first with TGF‐β1 to induce EMT and then with different concentrations of NC (2.5, 5.0, 7.5 and 10.0 μM) for 48 h. The expression levels of JAK2, p‐JAK2, STAT3 and p‐STAT3 were then detected by western blotting. (C, D) Quantitative analysis of the protein levels, Data were obtained from three independent experiments. *p<0.05, **p<0.001, ***p<0.0001

The results described above clearly demonstrate that NC can inhibit EMT in glioma cells, but the underlying mechanism remains unknown. Recent research has shown that the JAK2/STAT3 pathway plays a key role in tumorigenesis and progression of a variety of tumor types.^20^ By controlling the expression of tumor‐related genes, this pathway can regulate the tumor cell cycle and apoptosis, promoting tumor metastasis and immune escape.^21^ We detected the effect of NC on JAK2/STAT3 signaling by western blot analysis of related proteins in NC‐treated glioma cells. The experimental results exhibited that NC treatment inhibited the expression of JAK2, p‐JAK2, STAT3, and p‐STAT3 in glioma cells, and these inhibitory effects increased with increasing NC concentration (Figure 7A and B).

### 3.7 Inhibition of JAK2/STAT3 pathway represses EMT in glioma cells

**FIGURE 8:**
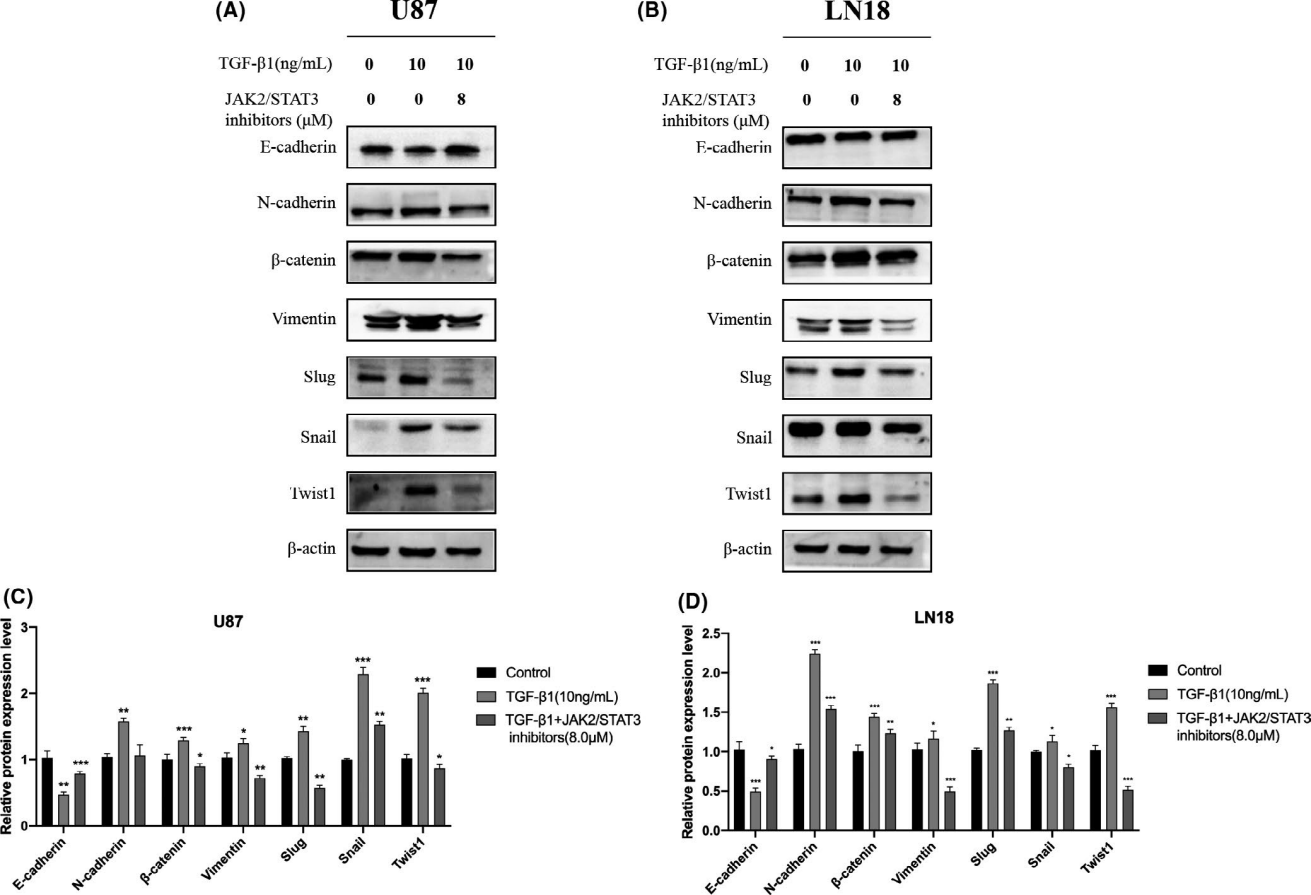
(A, B) U87 and LN18 glioma cells were treated with TGF‐β1 to induce EMT with and without 8 μM of the JAK2/STAT3 pathway inhibitor WP1066 for 48 h. Then the expression of EMT‐related marker proteins and transcription factors was detected by western blotting and compared with that in control cells. (C, D) Quantitative analysis of the protein levels, Data were obtained from three independent experiments. *p<0.05, **p<0.001, ***p<0.0001

WP1066 is a novel JAK2/STAT3 inhibitor that can selectively inhibit the JAK2/STAT3 signaling pathway. To clarify the role of JAK2/STAT3 signaling in glioma EMT and determine whether NC modulates JAK2/STAT3 signaling as a mechanism for inhibiting EMT in glioma cells, we used WP1066 to inhibit JAK2/STAT3 activity in U87 and LN18 glioma cells during exposure to TGF‐β1 for the induction of EMT. Changes in the expression of important markers of EMT were detected by western blotting. The experimental results showed that treatment with 8 μM WP1066 reduced the upregulation of EMT‐related mesenchymal markers N‐cadherin, β‐catenin, and vimentin, as well as transcription factors Twist1, Slug, and Snail, suggesting that the inhibition of STAT3 activity downregulated the expression of stromal tumor EMT‐related mesenchymal markers and transcription factors. These experimental results demonstrated that JAK2/STAT3 signaling was involved in the EMT process of glioma cells and suggested that the inhibitory effect of NC on glioma EMT may be achieved via the JAK2/STAT3 pathway (Figure 8A and B).

### 3.8 NC suppresses the self‐renewal capacity of glioma stem‐like cells

**FIGURE 9:**
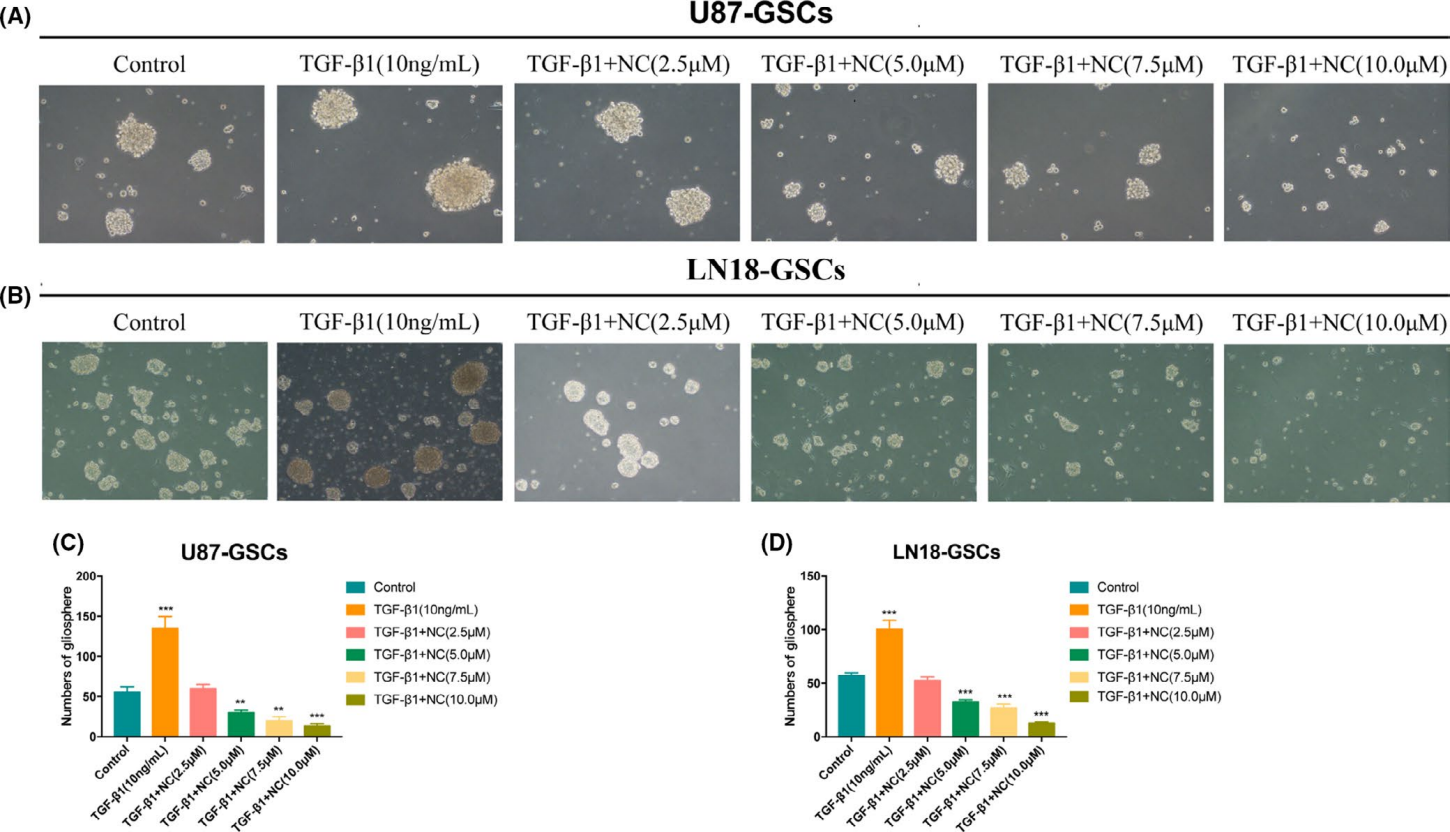
(A, B) Gliospheres were treated first with TGF‐β1 to induce EMT and then with different concentrations of NC (2.5, 5.0, 7.5 and 10.0 μM) for 48 h. Microscopic images (100x) of the gliospheres were taken, and the numbers of second‐generation gliospheres were counted. (C, D) Quantitative results for secondgeneration gliosphere formation after EMT induction and with and without NC treatment. Each independent experiment was repeated three times, and the results were compared with those of the control group. *p<0.05, **p<0.001, ***p<0.0001

The gliosphere formation assay is an important functional test for evaluating the self‐renewal capacities of cancer stem cells. Here we used the second‐generation gliosphere formation assay to detect the self‐renewal capacities of glioma stem‐like cells. The experimental results showed that the number of formed gliospheres after EMT induction was increased compared with that in the control group, and the number of formed gliospheres after NC treatment obviously decreased in a concentration‐dependent manner. These results indicated that the self‐renewal capacities of glioma stem‐like cells were enhanced after induced EMT, whereas NC treatment suppressed this enhanced self‐renewal capacity of glioma stem‐like cells after EMT (Figure 9A–D).

**FIGURE 10:**
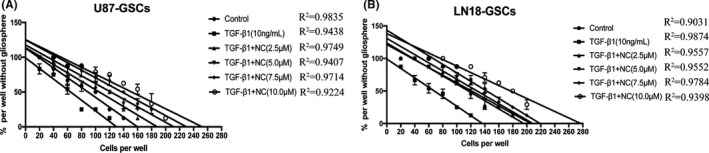
(A,B) Self‐renewal capacity of U87 and LN18 glioma stem‐like cells was detected by limited dilution assay after induction of EMT and after treatment with different concentrations of NC. The regression curve is shown, and the x‐intercept represents the number of cells required to form one gliosphere

The limiting dilution assay is an effective method for determining the proliferation of a single cancer stem cell. By analyzing the minimum number of cells required to form a tumor sphere, the frequency of tumor sphere formation among a tumor cell population can be quantitatively determined. The requirement of a smaller number of cells indicates a stronger self‐renewal capacity in tumor cells. The results showed that the self‐renewal capacity of U87 and LN18 glioma cells was enhanced after the induction of EMT. The minimum numbers of U87 and LN18 glioma cells required for gliosphere formation in the control groups were 160 and 195.8 per well, respectively. The numbers of U87 and LN18 glioma cells required for gliosphere formation after EMT induction were 130 and 136.9 per well, respectively. These numbers increased significantly (*p*<0.05) upon treatment with increasing concentrations of NC though (188.1 and 205.9, respectively, with 2.5 μM NC; 211.9 and 208.8, respectively, with 5.0 μM NC; 229.9 and 219.1, respectively, with 7.5 μM NC; and 252.4 and 277.3, respectively, with 10.0 μM NC). These results indicated that the EMT process enhanced the self‐renewal capacities of glioma stem‐like cells, whereas NC reduced the self‐renewal capacities of these cells (Figure 10A and B).

### 3.9 NC inhibits the enhanced expression of stem cell markers in glioma cells after EMT

**FIGURE 11:**
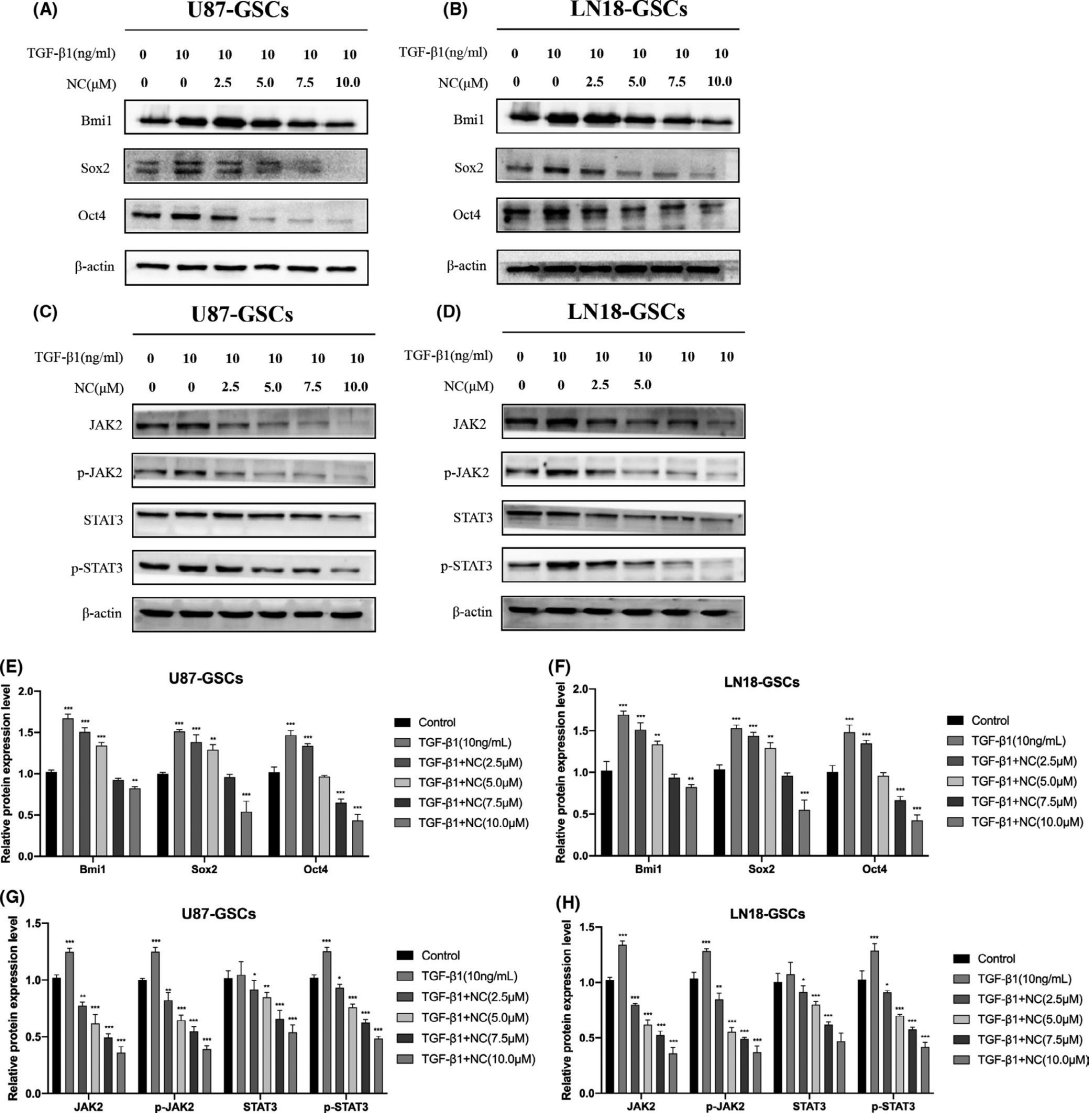
(A, B) After treatment of gliospheres with TGF‐β1 to induce EMT and then with various concentrations of NC, western blotting was performed to detect the expression levels of tumor stem cell markers Oct4, Bmi1, and Sox2. (C, D) After the same treatments of gliospheres, western blotting was also performed to detect the expression levels of JAK2, p‐JAK2, STAT3, and p‐STAT3. (E, F, G, H) Quantitative analysis of the protein levels, Data were obtained from three independent experiments. *p<0.05, **p<0.001, ***p<0.0001

We further studied whether EMT can promote the expression of cancer stem cell markers in glioma cells and whether NC has an inhibitory effect on the expression of these markers. Western blotting was used to detect the expression of cancer stem cell markers Oct4, Bmi1, and Sox2 after the induction of EMT in gliospheres as well as after treatment with NC (2.5, 5.0, 7.5, and 10.0 μM) for 48 h. Additionally, the changes in the expression levels of JAK2, p‐JAK2, STAT3, and p‐STAT3 pathway proteins were detected. β‐actin served as a protein loading control. The experimental results showed that the induction of EMT, indeed, increased the expression of stem cell markers in gliospheres. After NC treatment, the expression levels of JAK2, p‐JAK2, p‐STAT3, and STAT3 were reduced as were the expression levels of stem cell‐related markers Oct4, Bmi1, and Sox2, and these effects were concentration dependent (Figure 11A–D).

### 3.10 JAK2/STAT3 pathway inhibition suppresses the expression of glioma stem‐like cell markers

**FIGURE 12:**
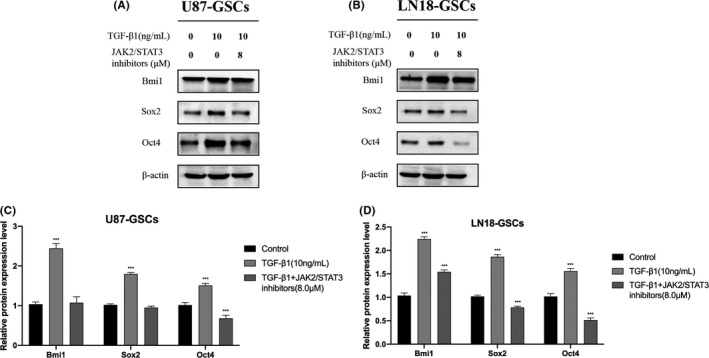
(A, B) U87 and LN18 glioma cells were treated with TGF‐β1 to induce EMT with and without 8 μM of the JAK2/STAT3 pathway inhibitor WP1066 for 48 h. Then the expression of stem cell marker proteins was detected by western blotting and compared with that in control cells. (C, D) Quantitative analysis of the protein levels, Data were obtained from three independent experiments. *p<0.05, **p<0.001, ***p<0.0001

To investigate whether NC inhibits the expression of stem cell‐related markers through the modulation of JAK2/STAT3 signaling, we used WP1066 to inhibit JAK2/STAT3 signaling and further studied the effect of JAK2/STAT3 activity on the expression of stem cell markers in glioma cells. The experimental results showed that treatment with 8 μM WP1066 reduced the expression of stem cell‐related markers Oct4, Bmi1, and Sox2 in glioma cells after EMT. These results indicated that JAK2/STAT3 signaling was involved in the expression of Bmi1, Sox2, and Oct4 by glioma cells. Moreover, the inhibitory effect of NC in glioma cells may be achieved through altered JAK2/STAT3 pathway activity (Figure 12A and B).

## 4 DISCUSSION

Many studies showed that NC had a good inhibitory effect on the proliferation of glioma cells and gliospheres. Almost all malignant tumors have the characteristics of immortal proliferation, increased angiogenesis, and inhibition of apoptosis.^22^, ^23^ Cancer stem cells are closely related to the malignant progression of tumors and resistance to radiotherapy and chemotherapy. The ideal anti‐tumor drug should be able to effectively inhibit the proliferation of tumor cells, and this is the primary condition for the screening of anti‐tumor drugs. Prior research has shown that NC can inhibit tumor growth both in vivo and in vitro as well as inhibit the growth of cancer stem cells in a variety of tumor types.^9^, ^10^, ^11^, ^12^, ^24^ Our results indicate that NC has inhibitory effects on glioma cells and glioma stem‐like cells, suggesting the potential of NC as a new anti‐glioma drug.

NC reduced the expression of epithelial marker E‐cadherin in glioma cells, possibly by regulating the expression levels of transcription factors, and also increased the expression of mesenchymal markers N‐cadherin, vimentin, and β‐catenin, thereby inhibiting the EMT behavior of these cells. The epithelial cells obtain mesenchymal properties through EMT mainly by inhibiting the separation and formation of E‐cadherin and intercellular junctions.^25^ During the EMT process, the lysed E‐cadherin releases β‐catenin to become a transcriptional activator of cell proliferation. ^26^, ^27^ Decreased E‐cadherin expression in cancer cells is associated with tumor metastasis, invasion, and activation of various EMT‐related transcription factors.^28^ whereas N‐Cadherin can inhibit the EMT behavior of tumor cells. For example, N‐Cadherin in neural crest cells helps to establish strong adherent connections.^29^ Transcription factors such as Snail, the Twist family proteins, and the zinc finger family of proteins (E‐box‐binding homeobox, ZEB) can promote the EMT process of cells. Studies have shown that Snail, Slug, ZEB1, and ZEB2 can directly bind to the E‐box sequence in the promoter region of the E‐cadherin gene and inhibit its transcription.^30^, ^31^, ^32^, ^33^, ^34^ Vimentin is a cytoskeletal protein, and in the EMT process, vimentin plays an important role in changes in cell adhesion and movement.^35^, ^36^ However, from our results, the changes in the expression levels of EMT‐related proteins and transcription factors were not completely synchronized. This shows that NC has a certain reversal effect on the EMT behavior of glioma cells, but the process by which the cells transform from the epithelial state to the complete mesenchymal state is not an "all‐or‐none" transition and involves an intermediate state. "EMT intermediate state" refers to the gradual evolution of EMT from the beginning of EMT to the final completion of EMT, with some EMT markers expressed in the early stage of EMT and others showing altered expression in the later stage of EMT.^4^

Cancer stem cells in glioblastoma are closely related to EMT among tumor cells and resistance to radiotherapy and chemotherapy. In our experiment, NC showed a good inhibitory effect on the self‐renewal ability of glioma stem‐like cells in vitro and also reduced the expression of cancer stem cell markers Oct4, Sox2, and Bim1. Sox2, Oct4, and Bmi1 are effective markers of cancer stem cells, because they play important roles in the growth of cancer stem cells and promote the malignant progression of tumors.^37^, ^38^, ^39^, ^40^, ^41^, ^42^, ^43^, ^44^, ^45^, ^46^, ^47^, ^48^, ^49^, ^50^, ^51^ In esophageal squamous cell carcinoma and lung cancer.^52^ a significant correlation was found between Sox2 expression and patient survival and prognosis.^43^, ^44^ In neuroblastoma, Sox2 overexpression was shown to enhance tumorigenesis, while inhibiting tumor cell differentiation.^45^ Expression of Sox2 and Oct4 is positively correlated with the pathological grade of glioma.^46^ Elevated levels of Bmi1 are associated with a poor prognosis in patients with head and neck cancer, nasopharyngeal carcinoma, and glioma.^47^, ^48^, ^49^, ^50^, ^51^, ^52^, ^53^ These markers also show correlations with the proliferation of glioma stem cells and the resistance of gliomas to chemotherapy. For example, combined treatment with pentafluridol and temozolomide significantly inhibited tumor growth, while reducing the expression of Sox2 and Oct4 as well as the expression of EMT factors.^50^ The inhibitors of Bmi1 can effectively prevent the in vitro self‐renewal of glioblastoma stem cells and markedly extend the lifespan of mice with advanced disease.^51^ The inhibitory effects of NC on the expression of Oct4, Sox2, and Bim1 along with other stem cell markers in glioma may reduce the growth of glioma stem‐like cells and improve the survival rate of glioma patients.

In glioma, JAK2/STAT3 signaling may regulate the expression of E‐cadherin, N‐cadherin, vimentin, and β‐catenin via related transcription factors to inhibit the EMT process. Our results suggested that NC inhibited the related transcription factors through its effects on JAK2/STAT3 signaling and thereby inhibited EMT. NC also inhibited relevant glioma stem‐like cell‐related markers through its effects on the JAK2/STAT3 pathway. The EMT and cancer stem cells are inextricably linked, and they all play critical roles in the infiltration, metastasis, and recurrence of malignant tumors as well as tumor resistance to radiotherapy and chemotherapy. For example, in oral squamous cell carcinoma, the CCL21/CCR7 axis activates the JAK2/STAT3 signaling pathway to regulate the progression of EMT and promote the stemness of oral squamous cell carcinoma.^52^ In breast and ovarian cancer, the JAK2/STAT3 signaling pathway is related to the proliferation of cancer stem cells.^53^, ^54^ In advanced colorectal cancer, the JAK2/STAT3 signaling pathway can regulate the growth of cancer stem cells and even reverse the effects of radiotherapy, contributing to resistance to radiotherapy.^19^ As an effective JAK2/STAT3 inhibitor, NC represents a promising agent for the treatment of glioma.

Although NC can prevent the EMT behavior of glioma cells, application of NC in the clinical treatment of glioma patients will require more in‐depth research. The adverse effects of NC in vivo remain unclear and must be determined.

In conclusion, our results demonstrate that NC effectively inhibits the EMT process and cancer stem cell‐like properties in glioma cells and these effects of NC were achieved via JAK2/STAT3 signaling. These results indicate a potential clinical use of NC in the treatment of glioma and provide supporting evidence to warrant a further clinical trial for NC as an anti‐glioma drug.

## CONFLICTS OF INTEREST

The authors report no conflicts of interest.

## Data Availability

All data generated or analyzed during this study are included in this article.
